# Mechanism and effects of extramedullary hematopoiesis on anti-tumor immunity

**DOI:** 10.20892/j.issn.2095-3941.2023.0203

**Published:** 2023-07-24

**Authors:** Zefang Chen, Xinyu Cheng, Li Yang, Xiaoming Cheng, Bo Zhu, Haixia Long

**Affiliations:** 1 Institute of Cancer, Xinqiao Hospital, Third Military Medical University, Chongqing 400037, China; 2 School of Pharmacy and Bioengineering, Chongqing University of Technology, Chongqing 400054, China; 3 Department of Respiratory Disease, Xinqiao Hospital, Third Military Medical University, Chongqing 400037, China

Cancer immunotherapy is a preferred strategy for boosting anti-tumor immunity to eliminate malignant cells and is considered a major breakthrough in the field of cancer treatment; however, immunosuppressive cells in the tumor microenvironment (TME) pose a major obstacle to the efficacy of immunotherapy. Myeloid cells, such as tumor-associated macrophages (TAMs) and myeloid-derived suppressor cells (MDSCs), and immunosuppressive lymphocytes, such as regulatory T cells, are the most important immunosuppressive cells present within the TME. Such immunosuppressive cells are mainly produced by the orderly differentiation of stem and progenitor cells during bone marrow hematopoiesis. Nevertheless, recent studies have revealed that myeloid and erythroid progenitor cells (EPCs) generated by tumor-induced extramedullary hematopoiesis (EMH) have important roles in disrupting antitumor immunity and impairing the efficacy of immunotherapy. This review focuses on the mechanism underlying tumor-induced EMH, EMH-mediated immunosuppressive cell generation, and the effect on anti-tumor immunity (**Figure 1**).

**Figure 1:**
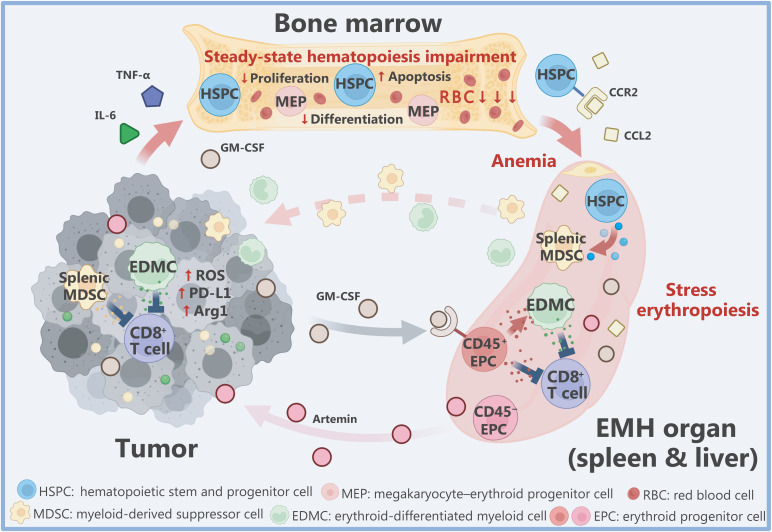
Extramedullary hematopoiesis (EMH) in cancer. Tumor-induced inflammatory factors, such as GM-CSF, TNF-α, and IL-6, damage bone marrow erythropoiesis and upregulate erythrocyte apoptosis, causing anemia and EMH. Hematopoietic progenitor cells (HSPCs) are recruited to the spleen through upregulated CCL2/CCR2 signaling and differentiate into splenic myeloid-derived suppressor cells (MDSCs) under the combined action of splenic environmental factors and endogenous to perform immunosuppressive functions. Tumor-induced EMH-generated CD45^−^ erythroid progenitor cells (EPCs) promote tumor progression by secreting artemin. The CD45^+^ EPC population inhibits proliferation and immune function of CD8^+^ T cells by promoting overexpression of ROS, PD-L1, and Arg1, thus disrupting antitumor immunity. Furthermore, daughter cell EDMCs mediated by tumor-induced GM-CSF signals establish stronger immunosuppressive functions. (The figure was created with BioRender.com)

## EMH in cancer

Mammalian hematopoietic activity begins in the yolk sac and moves to the fetal liver and spleen to produce blood cells as needed^1^. After birth, the bone marrow takes over the responsibility of providing and replenishing blood cells, and hematopoietic activity in the liver and spleen ceases. Hematopoietic progenitor cells (HSPCs) in the bone marrow with the capacity for self-renewal and differentiation are produced and continuously replenish all cellular components of the blood system^2^. Traditionally, HSPCs develop into the lymphoid, myeloid, and erythroid lineages, and the probability of developing into each lineage is relatively stable. In the physiologic state, there is no apparent intersection that differentiates between lineages, thus maintaining a lineage balance in the blood system.

EMH, however, occurs in pathologic conditions, such as infections, metabolic stress, anemia, and cancer, thus leading to impaired hematopoietic balance in the bone marrow^3^. Among all the pathologic states that trigger EMH, tumor-associated anemia is particularly worth noting. Tumors are associated with the release of inflammatory factors, such as GM-CSF, tumor necrosis factor-α (TNF-α), and interleukin-6 (IL-6), which mainly cause inflammatory hypoferremia, iron-restricted erythropoiesis, and upregulated hemolysis, as well as decreased production and reactivity of erythropoietin (EPO)^4^. The mechanism underlying the release of inflammatory factors and the effects on EPO not only occurs in purely inflammatory conditions, but also due to the genetic regulatory aspects of erythropoiesis *via* GATA-1, GATA-2, IL-1 and interferon-γ^5^. Like many types of solid tumors, hematologic neoplasms, such as leukemia and multiple myeloma (MM), secrete inflammation factors that lead to disordered erythropoiesis and upregulated hemolysis. Specifically, among patients with MM, malignant plasma cells secrete monoclonal antibodies that target nearby bone tissue, which results in hypercalcemia, and together with myeloma-associated nephropathy caused by deposition of light chains in the distal convoluted tubule, a decreased production of EPO. In addition, unlike solid tumors in which infiltration is largely local before metastasis occurs, hematologic neoplasms impair the hematopoietic function directly by infiltrating the bone marrow. Indeed, abnormally proliferating, poorly differentiated cells invade and sabotage hematopoietic structures and microenvironment. Patients with hematologic neoplasms are also at high risk for anemia, but the underlying mechanisms are slightly different^6^. Because bone marrow hematopoiesis disorders lead to upregulation of erythrocyte apoptosis and phagocytosis, erythrocyte production declines rapidly and anemia occurs. Due to changes in the bone marrow microenvironment and the induction of cytokines which exist in anemia, the release of HSPCs from the bone marrow to the peripheral blood is much higher than in the physiologic state, and circulating HSPCs are then recruited to extramedullary organs, such as the spleen and liver^7^. When combined with changes in the local microenvironment, including hypoxia, a tumor-mediated systemic immune response, and large amounts of soluble factors, EMH niches form and activate extramedullary erythropoiesis^3,8^. EMH initiates erythropoiesis to compensate for the loss of bone marrow function when impaired bone marrow hematopoietic function is unable to produce sufficient blood cells; however, as a compensatory mechanism, EMH can only slightly compensate for bone marrow hematopoiesis dysfunction, which causes continuous progression and leads to EMH organ swelling. In most cases, EMH does not effectively alleviate the severity of tumor-associated anemia^9,10^. Although tumors promote EMH, the mechanism underlying EMH-mediated immunosuppressive cell generation and the role in anti-tumor immunity remain poorly understood.

## Splenic HSPC-mediated tumor-promoting myelopoiesis disrupts anti-tumor immunity

Recently, EMH-induced tumor-promoting myelopoiesis has been reported in the spleen of tumor-bearing mice, suggesting that the spleen is not only a reservoir of immune cells but also has a unique and important function in producing immunosuppressive myeloid cells, which are essential for tumor-induced anti-tumor immune tolerance. Using five transplanted or genetic tumor models, a previous study showed that in response to tumor-induced splenic EMH, splenic stromal cells of tumor-bearing mice recruited Lin^low/−^Sca-1^+^c-Kit^high^ (LSK) HSPC subpopulations from the peripheral circulation to the spleen *via* the CCL2/CCR2 axis^11^. Then, under the “education” of soluble factors, such as IL-6 produced by splenic stromal cells and GM-CSF produced by CCR2-positive splenic LSK HSPCs, the myeloid commitment of HSPCs is synergistically driven to differentiate into highly immunosuppressive myeloid cells, which then inhibit T cell proliferation and antigen-specific cytotoxic activity. Researchers have specifically targeted splenic HSPCs with low-dose sorafenib, a c-kit inhibitor, to restrain splenic EMH while maintaining the physiologic function of the spleen. This treatment significantly induced apoptosis and inhibited proliferation of splenic HSPCs by reducing endogenous GM-CSF expression. Moreover, low-dose sorafenib treatment inhibited the suppressive effect of tumor MDSCs on T cell proliferation and cytotoxicity and synergistically enhanced the therapeutic efficacy of PD-L1 blockade.

Further studies demonstrated that most splenic LSK HSPCs with EMH showed signs of a strong endoplasmic reticulum stress response, including the activation of PKR-like endoplasmic reticulum kinase (PERK)^12^. HSPCs are then reprogrammed into committed MDSC precursors in the spleen *via* the PERK-ATF4-C/EBPβ signaling pathway. Similar to the splenic HSPC-targeted strategy of low-dose sorafenib, spleen-targeted PERK blockade restrains the immunosuppressive function of MDSCs and reshapes the tumor microenvironment, thus restoring anti-tumor immunity impaired by EMH and improving the efficacy of immunotherapy. This finding suggests that targeting EMH may be a novel strategy to enhance immunotherapy.

In addition, cancer-induced splenic accumulation of HSPCs has been observed in patients with different types of solid tumors, and patients with lower frequencies of CD133^+^ (a marker for human HSPCs) progenitors in the spleen had significantly longer survival than patients with higher frequencies^11^. These clinical results indicate that tumor-promoting myelopoiesis caused by splenic EMH has a significant negative effect on tumor patients and that CD133 can be used as a marker to predict the prognosis of patients with tumors.

## Tumor-associated EMH-induced erythroid-derived immunosuppressive cells impair antitumor immunity

In addition to the large accumulation of HSPCs in the spleen caused by tumor-induced EMH, a detailed analysis of cell composition revealed that EPCs are abundant aggregating cells in the spleen. This phenomenon is mainly caused by the obstructed differentiation of EPCs and is also found in other EMH organs, such as the liver^13^. CD45 has been reported to be a marker of early erythroid precursors that can distinguish EPCs at relatively early and late differentiation stages^14^. Several studies have reported the accumulation of both CD45^−^ and CD45^+^ EPCs in the spleen; however, CD45^−^ and CD45^+^ EPCs have distinct mechanisms by which the EPCs are generated, as well distinct functional characteristics. Tumor-derived TGF-β contributes to the generation of splenic CD45^−^ EPCs, which promote tumor progression *via* the neurotrophic factor, artemin (ARTN). Increased expression of ARTN and its receptor is correlated with a poor prognosis in patients with different types of cancer^15^. Recombinant EPO promotes resistance to radiotherapy and anti-PD-L1 therapy by restoring the levels of CD45^−^ EPC and ARTN. Conversely, ARTN blockade augments the anti-tumor effects of anti-PD-L1 therapies in mice^15,16^.

Unlike CD45^−^ EPCs, CD45^+^ EPCs have been shown to produce large amounts of reactive oxygen species (ROS) that inhibit systemic anti-tumor immunity of CD8^+^ T cells, ultimately increasing the susceptibility of bacteria and viruses and weakening anti-tumor immunity^13^. More importantly, scRNA-seq data have revealed that tumor-derived CD45^+^ EPCs lose key transcription factors for erythroid differentiation, such as Gata1, Klf1, and Sox6, and gained transcription factors for myeloid differentiation (i.e., PU.1), suggesting that CD45^+^ EPCs may have myeloid differentiation potential. Further research has revealed that GM-CSF produced by tumor cells mediates CD45^+^ EPCs trans-differentiation into the myeloid lineage and generates an erythroid-myeloid hybrid cell population, the erythroid-differentiated myeloid cells (EDMCs)^17^. Functional studies have shown that terminal differentiation of CD45^+^ EPCs into EDMCs achieves a wider variety of robust inhibition of T cell activation and attenuates the efficacy of anti-PD-L1 treatment by upregulating PD-L1, ICOS-L, NOX2, and Arg1 and generating high levels of TGF-β and IL-10. Clinically, high infiltration of intratumoral EDMCs is consistent with T cell exhaustion and an immunosuppressive microenvironment. Additionally, high infiltration of EDMCs is negatively correlated with the therapeutic efficacy of PD-1/PD-L1 antibodies in patients with cancer.

Another clinical study reported that CD45^+^ CD71^+^ erythroid cells accumulate in the peripheral circulation and tumor tissues of patients with hepatocellular carcinoma (HCC)^18^. These cells exhibit stronger suppressive functions by producing ROS, IL-10, and TGF-β, and the degree of infiltration can be used to predict the disease-free survival and overall survival of patients with HCC. Researchers have concluded that the number of CD45^+^ CD71^+^ erythroid cells in tumor tissues may be a reliable biomarker for HCC recurrence after radical surgery.

## Tumor-associated anemia as a marker for EMH effectively predicts efficacy of PD-1/PD-L1 blockade

Tumor-associated anemia is the most common complication in > 30% of patients with cancer who are diagnosed prior to the initiation of antineoplastic therapy, especially in patients with advanced tumors. Between 40% and 70% of patients who are not anemic at the time of diagnosis also develop anemia after cancer treatment, which has a significant impact on survival, disease progression, treatment, and quality of life^19,20^. With respect to the mechanism by which tumor-associated anemia leads to a poor prognosis, it is currently thought that anemia aggravates hypoxia and promotes the Warburg effect, thus impairing antitumor immunity and reducing the efficacy of chemotherapy and radiotherapy. With the discovery that anemia triggers EMH not only by failing to replenish red blood cells, but also in generating immunosuppressive cells, such as splenic MDSCs and erythroid-derived immunosuppressive cells, anemia is widely recognized for its ability to inhibit antitumor immunity.

Recent analyses of cohorts of patients with various types of solid tumors who received PD-1/PD-L1 blockade showed that anemia is strongly associated with poor prognosis in most types of cancers^17^. Compared to patients with mild or normal hemoglobin (Hb) levels, patients with moderate-to-severe anemia (Hb < 90 g/L) have significantly shorter progression-free and overall survival. Moreover, no partial responses have been observed in patients with anemia and the best outcome is stable disease. As mentioned above, the deletion or inhibition of EMH-induced immunosuppressive cells significantly boosts the PD-L1 blockade effect^11^. Hence, the severity of tumor-associated anemia may serve as a potential predictor for the prognosis of patients with cancer and the therapeutic effect of immune checkpoint inhibitors, especially PD-1/PD-L1 blockade.

## Summary

Tumor-induced signals and changes in the tissue microenvironment initiate tumor-induced EMH, which produces multiple populations of immunosuppressive cells, not only by promoting myelopoiesis of EMH-induced splenic HSPCs^11,12^, but also by accumulating EPCs in the spleen and mediating CD45^+^ subset differentiation into EDMCs^13,15–17^. These EMH-related immunosuppressive cells induce anti-tumor immune damage and immunotherapeutic resistance by impairing the proliferation and immune functions of CD8^+^ T cells. The deletion or inhibition of these cells reverse immunosuppression and improve the efficacy of immunotherapy. As a critical factor in EMH initiation and acceleration, anemia is also strongly correlated with the immunosuppressive status of patients and could be a valuable predictor of prognosis and immunotherapy efficacy.

## Discussion

Researchers on the mechanisms of tumor-associated EMH and its effects on anti-tumor immunity have enhanced our knowledge of the hematopoietic system in tumor conditions; however, the underlying mechanisms that induce tumor-associated EMH and mediate EMH-associated immunosuppressive cell generation remain largely unclear.

Many clinical trials have reported that anemia is one of the most common complications of malignancies, for which there are numerous clinical management strategies. The usual management of anemia focuses on stimulating the proliferation and differentiation of progenitor cells, especially EPCs, by stimulators, such as EPO or EPO analogs, and supplying nutrition needed because anemia is mainly caused by dysfunction of bone marrow hematopoiesis and upregulated hemolysis. In the case of tumor-associated anemia, EPO stimulates erythropoiesis while the differentiation of EPCs is still obstructed, which can lead to more severe accumulation of EPCs and splenomegaly, followed by further EMH, immunosuppression, and resistance to the PD-L1 blockade^15^. In this case, remitting EMH and simultaneously correcting the differentiation behavior of HSPCs and EPCs which have accumulated in the EMH organs are more reasonable approaches.

Furthermore, current strategies to improve the efficacy of immunotherapy mainly involve the intervention of immunosuppressive cells, such as MDSCs and regulatory T cells. The effects of the current strategies are somewhat limited and conserved, suggesting that there are unknown cells that establish immunosuppressive functions to disrupt immunotherapeutic efficacy. Recent studies have revealed the existence and function of special immunosuppressive cells induced by tumor-associated EMH, as well as the possibility of reversing EMH progression and suppression. These findings provide new prospects for relieving resistance to anti-tumor immunity and improving immunotherapeutic efficacy. Therefore, apart from familiar immunosuppressive cells, attention should be paid to tumor-associated EMH, which promotes myelopoiesis and erythropoiesis. In conclusion, strategies for reversing tumor-associated EMH and targeting immunosuppressive cells generated by tumor-associated EMH should be developed based on the generation mechanisms and molecular signatures of these cells.

## References

[1] Medvinsky A, Rybtsov S, Taoudi S (2011). Embryonic origin of the adult hematopoietic system: advances and questions. Development.

[2] Mazo IB, Massberg S, von Andrian UH (2011). Hematopoietic stem and progenitor cell trafficking. Trends Immunol.

[3] Yang X, Chen D, Long H, Zhu B (2020). The mechanisms of pathological extramedullary hematopoiesis in diseases. Cell Mol Life Sci.

[4] Dulmovits BM, Tang Y, Papoin J, He M, Li J, Yang H (2022). HMGB1-mediated restriction of epo signaling contributes to anemia of inflammation. Blood.

[5] Dicato M, Plawny L, Diederich M (2010). Anemia in cancer. Ann Oncol.

[6] Xu Z, Huang X (2021). Cellular immunotherapy for hematological malignancy: recent progress and future perspectives. Cancer Biol Med.

[7] Sohawon D, Lau KK, Lau T, Bowden DK (2012). Extra-medullary haematopoiesis: a pictorial review of its typical and atypical locations. J Med Imaging Radiat Oncol.

[8] Johns JL, Christopher MM (2012). Extramedullary hematopoiesis: a new look at the underlying stem cell niche, theories of development, and occurrence in animals. Vet Pathol.

[9] Fan N, Lavu S, Hanson CA, Tefferi A (2018). Extramedullary hematopoiesis in the absence of myeloproliferative neoplasm: Mayo clinic case series of 309 patients. Blood Cancer J.

[10] Rashidi A, Courville E (2019). Massive splenomegaly. N Engl J Med.

[11] Wu C, Ning H, Liu M, Lin J, Luo S, Zhu W (2018). Spleen mediates a distinct hematopoietic progenitor response supporting tumor-promoting myelopoiesis. J Clin Invest.

[12] Liu M, Wu C, Luo S, Hua Q, Chen HT, Weng Y (2022). Perk reprograms hematopoietic progenitor cells to direct tumor-promoting myelopoiesis in the spleen. J Exp Med.

[13] Zhao L, He R, Long H, Guo B, Jia Q, Qin D (2018). Late-stage tumors induce anemia and immunosuppressive extramedullary erythroid progenitor cells. Nat Med.

[14] Harashima A, Suzuki M, Okochi A, Yamamoto M, Matsuo Y, Motoda R (2002). CD45 tyrosine phosphatase inhibits erythroid differentiation of umbilical cord blood CD34+ cells associated with selective inactivation of lyn. Blood.

[15] Hou Y, Liang HL, Yu X, Liu Z, Cao X, Rao E (2021). Radiotherapy and immunotherapy converge on elimination of tumor-promoting erythroid progenitor cells through adaptive immunity. Sci Transl Med.

[16] Han Y, Liu Q, Hou J, Gu Y, Zhang Y, Chen Z (2018). Tumor-induced generation of splenic erythroblast-like ter-cells promotes tumor progression. Cell.

[17] Long H, Jia Q, Wang L, Fang W, Wang Z, Jiang T (2022). Tumor-induced erythroid precursor-differentiated myeloid cells mediate immunosuppression and curtail anti-PD-1/PD-l1 treatment efficacy. Cancer Cell.

[18] Chen J, Qiao YD, Li X, Xu JL, Ye QJ, Jiang N (2021). Intratumoral CD45(+)CD71(+) erythroid cells induce immune tolerance and predict tumor recurrence in hepatocellular carcinoma. Cancer Lett.

[19] Steinberg D (1989). Anemia and cancer. CA Cancer J Clin.

[20] Oh DY, Lee KH, Lee DW, Yoon J, Kim TY, Bang JH (2022). Gemcitabine and cisplatin plus durvalumab with or without tremelimumab in chemotherapy-naive patients with advanced biliary tract cancer: an open-label, single-centre, phase 2 study. Lancet Gastroenterol Hepatol.

